# Epipericardial fat necrosis: increasing the rate of diagnosis by disseminating knowledge within a single institution

**DOI:** 10.1590/0100-3984.2016.0095

**Published:** 2018

**Authors:** Karina de Souza Giassi, André Nathan Costa, Ronaldo Adib Kairalla, José Rodrigues Parga Filho

**Affiliations:** 1 Faculdade de Medicina da Universidade de São Paulo (FMUSP), São Paulo, SP, Brazil

Dear Editor,

Epipericardial fat necrosis (EFN), an inflammatory process that occurs within the epipericardial fat and leads to encapsulated fat necrosis, has long been described as a rare entity^1^. However, since 2012-when the first case was reported in Brazil^2^-the number of reports have been increasing worldwide. In fact, there were only 23 cases reported between 1957 and 2010, in comparison with 26 new cases reported between 2011 and 2015^3^. What could explain this increase? Analyzing the data from a retrospective analysis of EFN at a quaternary hospital in the city of São Paulo, Brazil, and its impact on the diagnosis of the entity, we have made some assumptions.

**Figure 1:**
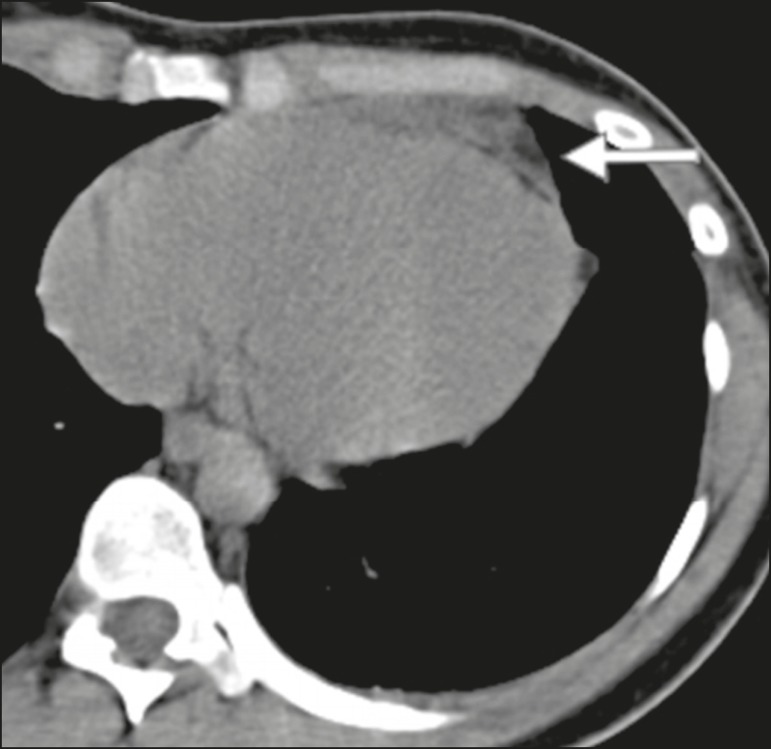
CT scan of a 29-year-old female with acute pleuritic chest pain showing a soft, round, fatty attenuating lesion in the epipericardial fat, the pain and the lesion both being features that are characteristic of EFN.

From 2011 to 2014, 20 cases of EFN were diagnosed on the basis of chest computed tomography (CT) scans performed in the emergency department (ED) of our institution. That was the focus of a previous retrospective analysis^1^, in which 11 cases of EFN were initially described from 3604 CT scans analyzed by two thoracic radiologists^1^. Scans were considered positive for EFN-described as "a soft, round, fatty attenuating lesion in the epipericardial fat, with or without pericardial thickening"^4^, as depicted in Figure 1-if both radiologists agreed. The authors of a case series analyzing previous reports suspected that EFN is, in fact, an underdiagnosed condition, and a subsequent study retrospectively analyzed 7463 CT scans, comparing clinical and laboratory data of the patients with those of control subjects^3^. The study described 20 cases and reported the incidence of EFN in ED patients with acute atypical chest pain^5^ to be 2.15% at the institution.

In 2013, the radiology department of our institution decided to disseminate information regarding the clinical and radiological features of EFN, in order to make radiologists aware of the entity, which was formerly considered to be extremely uncommon. The information was disseminated by the presentation of cases and lectures in multidisciplinary meetings, as well as in meetings of the radiology residence program. The radiological features of EFN were also presented to the radiologists of the ED. The data of the study were further analyzed in order to determine whether the radiologist had previously diagnosed the entity correctly in the formal report.

All 20 reports were reviewed and defined as follows: "not described"-when features of EFN were overlooked; "described but not suggested"-when EFN features were described but the diagnosis was not suggested in the report; and "suggested"-when EFN findings were described and its diagnosis was suggested.

**Figure 2:**
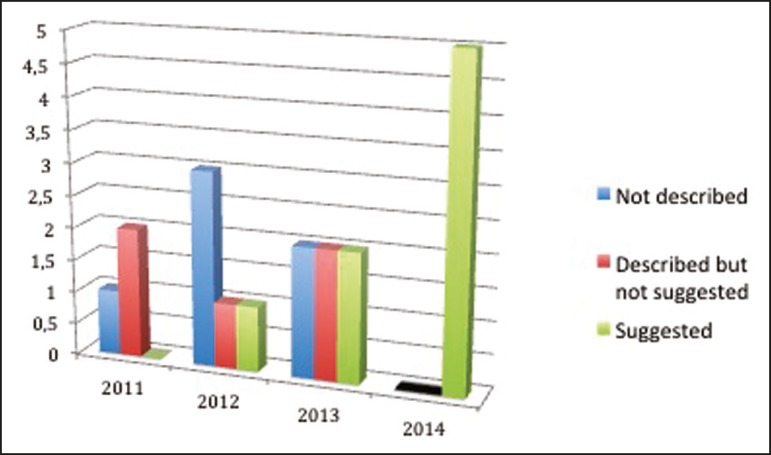
The 4-year progression of EFN diagnosis data at a single institution.

The outcome was surprising. As shown in Figure 2, we found a progressive number of diagnoses over the years, especially after 2013. In 2011, when radiologists were still unaware of the entity, the number of correct diagnoses was zero. In 2014, after the educational intervention, there were no more missed diagnoses of EFN at the institution.

We can suggest that the dissemination of knowledge at our institution changed the pattern of the diagnosis of a disease. We believe that, in the next few years, EFN will become known worldwide, the labels "rare" and "unknown" therefore no longer being associated with this entity.
